# Acute Toxicity, Phytochemical Screening, Analgesic, and Anti-Inflammatory Activities of Aqueous and Methanol Root Extracts of *Maerua triphylla* A. Rich. (Capparaceae)

**DOI:** 10.1155/2021/3121785

**Published:** 2021-11-15

**Authors:** Brian Muyukani Wangusi, Laetitia Wakonyu Kanja, Isaac Mpapuluu Ole-Mapenay, Jared Misonge Onyancha

**Affiliations:** ^1^Department of Public Health, Pharmacology and Toxicology, College of Veterinary and Agricultural Sciences, University of Nairobi, P.O. Box 29053-00625, Nairobi, Kenya; ^2^Department of Pharmacognosy, College of Health Sciences, Mount Kenya University, P.O. Box 342-01000, Thika, Kenya

## Abstract

*Maerua triphylla* root extracts are used by Maasai and Kikuyu communities in Kenya to manage headaches, stomachaches, migraines, and rheumatism. However, scientific data on their safety and efficacy are limited. The current study aims to investigate the safety, phytochemical constituents, analgesic, and anti-inflammatory activities of *M. triphylla* root extracts. Aqueous and methanol *M. triphylla* root extracts were prepared by cold maceration, and the extracts' safety was evaluated using *Wistar* rats according to the Organization for Economic Cooperation and Development (2008) guidelines. Standard qualitative phytochemical screening methods were used for the detection of various phytochemical groups in the extracts. Analgesic activity assay in *Swiss albino* mice was done using the acetic acid-induced writhing test, while anti-inflammatory activity was determined in *Wistar* rats using the acetic acid-induced paw edema method. The methanol and aqueous extracts revealed LD_50_ > 2000 mg/kg bw, classifying them as nontoxic. The presence of cardiac glycosides, flavonoids, alkaloids, and phenols was observed in both extracts. However, saponins were only present in the methanol extract. In the analgesic study, mice that received 100 mg/kg bw and 500 mg/kg bw of aqueous root extract of *M. triphylla* had significantly lower acetic acid-induced writhing than mice that received acetylsalicylic acid 75 mg (reference drug) (*p* < 0.05). Additionally, mice that received 500 mg/kg bw of methanol root extract of *M. triphylla* had significantly lower acetic acid-induced writhing than mice that received the acetylsalicylic acid 75 mg (*p* < 0.05). In the anti-inflammatory study, there was no significant difference (*p* < 0.05) between the inhibitory activity of different doses of the aqueous root extract of *M. triphylla* and a 50 mg/kg dose of diclofenac sodium (reference drug) on acetic acid-induced paw edema in rats. Moreover, there was no significant difference in the inhibitory activity of 100 mg/kg bw and 500 mg/kg bw doses of the methanol root extract of *M. triphylla* and a 50 mg/kg dose of diclofenac sodium on acetic acid-induced paw edema (*p* > 0.05). These findings suggest that the roots of *M. triphylla* may be useful in the safe mitigation of pain and inflammation and therefore support their ethnomedicinal use in the management of pain and inflammation.

## 1. Introduction

Pain is an unwanted emotional or receptive sensation localized to a part of the body. It is often described in terms of penetrative or tissue-destructive process like stabbing, burning, tearing, and squeezing [1]. Bradykinin, prostaglandins, and histamine are the major mediators of pain. On the other hand, inflammation is the body's normal, protective response to tissue injury caused by physical trauma, noxious chemicals, or microbiologic agents, which is a part of the host defense [2]. Inflammation usually subsides on completion of the healing process, but sometimes it turns to be severe and may be fatal, leading to diseases such as arthritis, rheumatism, and cancer [3, 4]. The treatment of such diseases generally relies on a large number of commercial preparations, including nonsteroidal anti-inflammatory drugs (NSAIDs), corticosteroids, and opioid analgesics [5]. However, most of these drugs have adverse effects like peptic ulcer, dyspepsia, and gastrointestinal bleeding [6]. Furthermore, these drugs are costly and have low efficacy [7]. Therefore, a large number of medicinal plants need to be investigated for their alternative use as analgesic and anti-inflammatory agents.

*M. triphylla* (Capparaceae) is an evergreen shrub growing to a height of up to 3 meters above ground. The roots of *M. triphylla* have been used to treat conditions like rheumatism, headache, migraine, diarrhea, and stomachache by the Maasai and Kikuyu communities in Kenya [8]. However, there is insufficient scientific data to support this ethnomedicinal use of *M. triphylla* roots in management of pain and inflammatory conditions. Moreover, the safety of using the root extracts from *M. triphylla* is unknown [8]. The current study assessed acute oral toxicity, phytochemical groups, pain-relieving, and anti-inflammatory properties of *M. triphylla* root extracts.

## 2. Materials and Methods

### 2.1. Plant Materials

Fresh roots of *M. triphylla* were obtained from Kajiado County, Ilbisil area, with the assistance of a renowned local herbalist. Identification and authentication of the prepared specimen were done by a plant taxonomist at the Department of Land Resource Management and Agricultural Technology (LARMAT), University of Nairobi. The voucher specimen number LARMATCAP36 was assigned to the specimen. The collected roots of *M. triphylla* were cleaned with running water, cut into small pieces, air-dried in a well-ventilated room for 14 days, and crushed into coarsely powdered material using an electric grinder. The powder was kept in a well-labeled manila sack and kept in a cool and nonhumid place awaiting extraction.

### 2.2. Extraction Methods

#### 2.2.1. Methanol Extract

Extraction was done according to the procedure described in [9] and later modified in [10]. In summary, 250 g of the root powder was soaked in 1 liter of analytical grade methanol using a 1.8-liter conical flask and then covered with a foil paper with constant shaking for 48 hours to macerate. This procedure was repeated for another batch of 250 grams of the root powder. The resultant mixture was then decanted and filtered through a cotton gauze, and the filtrate was further filtered through a Whatman No. 1 filter paper. The resultant filtrates were combined and reduced in vacuo at 50°C using a rotary evaporator. Thereafter, the extract was transferred into a clean, dry, and light-resistant glass bottle and placed in a sand bath set at 35°C to further remove the solvent and concentrate the extract. The actual weight of the dried extract was measured using an analytical balance and recorded before it was stored at 4°C in a refrigerator pending biological assay.

#### 2.2.2. Aqueous Extract

About 250 g of the *M. triphylla* root powder was cold macerated in 1.25 liters of distilled water in a 1.8-liter conical flask and then covered with a foil paper with constant shaking for 24 hours. This procedure was repeated for another batch of 250 g of the root powder. The resulting mixtures were decanted and filtered through a Whatman No. 1 filter paper. The resultant filtrates were then lyophilized using a freeze-dryer. The obtained freeze-dried product was weighed using an analytical balance, and the weight was recorded before being stored in tightly closed, light-resistant bottles at 4°C in a refrigerator pending biological assay [10].

### 2.3. Experimental Animals

In this study, both male and female *Wistar* rats (8–10 weeks old, weighing 110 ± 20 g) obtained from the animal house of the Department of Public Health, Pharmacology and Toxicology (PHPT) of the University of Nairobi (UoN) were used to assess the acute oral toxicity and anti-inflammatory activities of *M. triphylla* root extracts. Both male and female *Swiss albino* mice (4-5 weeks old, weighing 30 ± 5 g) obtained from the animal breeding facility at Veterinary Farm Kabete were used to assess the analgesic activities of *M. triphylla* root extracts. The experimental mice were delivered to the animal house of the department of PHPT of the University of Nairobi (UoN) and were allowed to acclimatize for seven days before the beginning of the experiment.

All the experimental animals were nulliparous and nonpregnant. The animals were housed in polypropylene cages in standard laboratory conditions. Tap water and standard laboratory animal pellets were provided ad libitum. Animal use and care guidelines outlined by the Faculty of Veterinary Medicine Biosafety, Animal Use and Ethics Committee (BAUEC) of the University of Nairobi and the National Council for Science, Technology and Innovation (NACOSTI) were adhered to in this study.

### 2.4. Preparation of Administration Doses

In this study, the Organization for Economic Cooperation and Development [11] (Document No. 425) standards in [12] were followed in the preparation of the doses for administration. Shortly, to make a stock solution with a dosage level of 500 mg/kg bw for administration to a rat weighing 100 g, the following formula described in [12] was followed:

(1) $$\begin{array}{c} \text{animal dose}\left(\text{mg}/\text{kg}\text{ bw}\right)=\frac{\text{bodyweight of the animal}\left(\text{g}\right)\times \text{selected dose}}{1000\text{ g}}\text{.} \end{array}$$

Therefore, animal dose(mg/kg bw)=(100 g/1000 g) × 500 mg=50 mg.

In accordance with the Organization for Economic Cooperation and Development [11] (Document No. 425) guidelines, 50 mg should be reconstituted in 0.2 ml of the physiological saline (vehicle). In this study, a 10 ml stock solution containing 500 mg/kg bw of either the aqueous or the methanol root extracts of *M. triphylla* was prepared and serially diluted with physiological saline to generate dosages of 100 mg/kg bw and 20 mg/kg bw doses. Similarly, this procedure was followed for the reference drug.

### 2.5. Acute Oral Toxicity Effects of *M. triphylla* Aqueous and Methanol Extracts

To investigate the safety of the aqueous and methanol extracts of *M. triphylla*, the Up-and-Down procedure for acute oral toxicity described by Organization for Economic Cooperation and Development [11] (Document No. 425) was used. The female *Wistar* rats were randomly assigned to three groups of five (5) rats each. Each animal was individually weighed and labeled with a permanent marker on its tail. They fasted overnight before the commencement of the study. The limit test was conducted by administering an oral single dose of 2000 mg/kg bw aqueous and methanol root extract of *M. triphylla* to Group I and Group II, respectively. Group III was treated as negative control and was administered with physiological saline orally. Thereafter, wellness parameters such as mortality, lethargy, salivation, mucous membrane appearance, skin, hair, diarrhea, unconsciousness, changes in body weight, and sleep were observed and documented in 30 minutes, 1 hour, 4 hours, 24 hours, 48 hours, 7 days, and 14 days correspondingly.

### 2.6. Phytochemical Screening of *M. triphylla* Root Aqueous and Methanol Extracts

The presence of common groups of phytoconstituents was detected in *M. triphylla* root aqueous and methanol extracts using the methods described by Harborne [13] and Kokate et al. [14] with modification. Froth test was used to detect saponins and Dragendorff's test for alkaloids; the flavonoids were revealed by the alkaline reagent test. Ferric chloride test was used to evaluate the presence of phenolic compounds and tannins. The existence of cardiac glycosides, steroids, and terpenoids was assessed by the Keller-Kilian test for cardiac glycosides and the Salkowski test was used for steroids and terpenoids.

### 2.7. In Vivo Analgesic Activity of *M. triphylla* Root Aqueous and Methanol Extracts

The analgesic effects of the aqueous and methanol root extracts of *M. triphylla* were evaluated using the acetic acid-induced writhing procedure developed by [15] in *Swiss albino* mice of either sex. The mice were randomly assigned to five groups (V, W, X, Y, and Z), with each group consisting of 5 animals. Groups V and W received 300 *µ*l physiological saline and 75 mg/kg bw acetylsalicylic acid orally as negative and positive controls, respectively. Groups X, Y, and Z on the other hand received an oral treatment of 20 mg/kg bw, 100 mg/kg bw, and 500 mg/kg bw, respectively, of the aqueous and methanol root extracts of *M. triphylla*. After a half-hour, writhing was induced in each mouse with a 200 *µ* 0.6% v/v acetic acid injection intraperitoneally.

Five minutes after writhing induction, the animals were individually observed and counting of the number of writhes was done for a half-hour and documented. The mean count of writhes and the percentage inhibition of writhing was determined as an index of analgesic activity using the formula defined by Rashid et al. [15]:

(2) $$\begin{array}{c} \text{percentage writhing inhibition}=\frac{\left(W_{c}-W\right)}{W_{c}}\;\times 100, \end{array}$$

where *W*_*c*_ is the mean number of writhes in the negative control group and *W* is the mean number of writhes in the positive control and experimental group.

### 2.8. Determination of the Anti-Inflammatory Effects of *M. triphylla* Root Aqueous and Methanol Extracts

The anti-inflammatory activity of *M. triphylla* root extracts was evaluated using 0.6% v/v acetic acid as the inflammation-inducing agent and diclofenac sodium as the reference drug. The experimental rats of either sex were randomly divided into five groups (V, W, X, Y, and Z), with each group having 5 rats. Evaluation of the anti-inflammatory activity was carried out as described by Winter et al. [16]. The diameter of the intact rat's right hind paw was measured in millimeters in all groups using a digital vernier caliper and recorded. Groups V and W received 1.5 ml physiological saline and 50 mg/kg bw diclofenac sodium orally as negative and positive controls, respectively. Groups X, Y, and Z on the other hand received an oral treatment of 20 mg/kg bw, 100 mg/kg bw, and 500 mg/kg bw, respectively, of *M. triphylla* root aqueous and methanol extracts. Two hundred microliters of 0.6% v/v acetic acid was then injected into the subplantar tissue of the right hind paw 30 minutes after administration of the treatments to induce edema.

After inflammation induction, the diameter of the right hind paw was measured hourly from the first hour up to the fifth hour. Paw diameter measured prior to the acetic acid injection was then compared with the diameter of the same paw after acetic injection by calculating the percentage inhibition and percentage change using the formula described by Omowumi et al. [17]:

(3) $$\begin{array}{c} \text{percentage inhibition of oedema}=\frac{\left(T-T_{0}\right)}{T}\times 100, \end{array}$$

where *T* is the thickness of paw in the negative control group and *T*_0_ is the thickness of paw in the experimental and negative control group.

### 2.9. Statistical Data Management and Analysis

The data obtained from analgesic and anti-inflammatory activities were tabulated on Microsoft Excel spreadsheet (2016), expressed as Mean ± Standard Error of the Mean (SEM), and analyzed using analysis of variance (ANOVA) and the two-sample *t*-test by GenStat statistical software 4th edition. This was followed by Tukey's post hoc test for pairwise comparison and separation of means at *α* = 0.05. Values with *p* ≤ 0.05 were considered statistically significant. Acute oral toxicity data were quantitatively and qualitatively analyzed according to Organization for Economic Cooperation and Development [11] guidelines (Document No. 425), and the LD_50_ value was recorded.

### 2.10. Ethical Considerations

Permission to conduct the current study was obtained from the Faculty of Veterinary Medicine Biosafety, Animal Use and Ethics Committee (BAUEC) of the University of Nairobi with a permit reference number FVM BAUEC/2021/291. The research license for the study was also obtained from the National Commission for Science, Technology, and Innovation (NACOSTI) with a license number NACOSTI/P/21/9494.

## 3. Results

### 3.1. Acute Oral Toxicity Effects of *M. triphylla* Root Extracts

The observations from acute oral toxicity studies of aqueous and methanol *M. triphylla* root extracts revealed the absence of signs of toxicity and lethal effects in *Wistar* rats at the limit/cut-off dose of 2000 mg/kg bw. The doses that could be lethal to half of the experimental rats (LD_50_ values) for *M. triphylla* root aqueous and methanol extracts were thus observed to be above 2000 mg/kg bw. Therefore, the extracts were classified as nontoxic according to the OECD 425 guidelines.

### 3.2. Phytochemical Screening

The results from the study indicated that *M. triphylla* root aqueous and methanol extracts contain alkaloids, flavonoids, cardiac glycosides, and phenolic compounds. Saponins were revealed only in methanol extract and were absent in the aqueous extract. Tannins, steroids, and terpenoids were not observed in both the aqueous and methanol extracts of *M. triphylla* root (Table 1).

### 3.3. In Vivo Analgesic Effects of the Aqueous and Methanolic Root Extracts of *M. triphylla*

Below is a summary of the effect of the aqueous and methanol root extracts of *M. triphylla* on acetic acid-induced writhing in mice. The effects are compared with those of 75 mg/kg bw dose of acetylsalicylic acid (reference drug).

In this study, the aqueous and methanol root extracts of *M. triphylla* inhibited acetic acid-induced writhing in mice in a dose-dependent manner (Table 2). However, there was no significant difference in the effect of a 75 mg/kg bw dose level of acetylsalicylic acid and a 20 mg/kg bw dose level of the aqueous *M. triphylla* root extract on acetic acid-induced writhing in mice (*p* > 0.05; Table 2). Conversely, the 100 mg/kg bw and 500 mg/kg bw dose levels of the aqueous root extract of *M. triphylla* produced a significantly higher inhibition of acetic acid-induced writhing than a 75 mg/kg bw dose level of acetylsalicylic acid (*p* < 0.05; Table 2).

On the other hand, the inhibition of acetic acid-induced writhing by a 75 mg/kg dose of acetylsalicylic acid was not significantly different from the inhibition resulting from the use of 20 mg/kg bw and 100 mg/kg bw dose levels of the methanol root extract of *M. triphylla* (*p* > 0.05; Table 2). However, the use of a 500 mg/kg bw dose level of the methanol root extract of *M. triphylla* produced a significantly higher inhibition of acetic acid-induced writhing than the use of 75 mg/kg bw dose level of acetylsalicylic acid (*p* < 0.05; Table 2).

**Figure 1:**
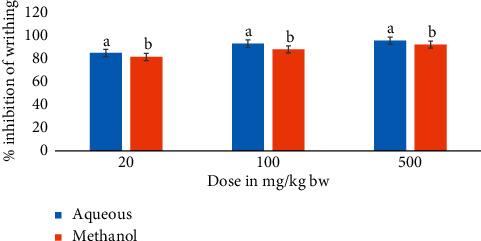
A comparison of the analgesic activities of aqueous and methanol root extracts of *M. triphylla* in *Swiss albino* mice. Bars are plotted as mean ± SEM. Bars with different superscript letters within the same dose level are significantly different (two-sample *t*-test; *p* < 0.05).

This study also included a comparison of *M. triphylla* root extract effects on acetic acid-induced writhing in mice (Figure 1).

The results showed that, at all dose levels, the mice that received the aqueous root extract of *M. triphylla* showed significantly higher percentage inhibition of the acetic acid-induced writhing than that recorded for mice that received the methanol root extract (*p* < 0.05; Figure 1).

### 3.4. In Vivo Anti-Inflammatory Effects of *M. triphylla* Root Aqueous and Methanol Extracts

#### 3.4.1. Effect of *M. triphylla* Root Aqueous and Methanol Extracts Treatment on Acetic Acid-Induced Paw Edema in *Wistar* Rats

Table 3 is a summary of the effect of aqueous and methanol root extracts of *M. triphylla* on acetic acid-induced paw edema in rats. The effects are compared with those of 50 mg/kg bw dose of diclofenac sodium (reference drug).

The results showed that there was no significant difference (*p* > 0.05) between the effect of different doses of the aqueous root extract of *M. triphylla* and a 50 mg/kg dose of diclofenac sodium on acetic acid-induced paw edema in rats (Table 3).

Conversely, the mice that received the methanolic root extract demonstrated significantly increased percentage inhibition of acetic acid-induced paw edema in rats in a dose-dependent fashion (*p* < 0.05; Table 3). A low dose of the methanol extract of *M. triphylla* produced a significantly lower (*p* < 0.05) inhibition of acetic acid-induced paw edema than a 50 mg/kg dose of diclofenac (Table 3). There was no significant difference in the inhibitory activity of intermediate and high doses of the methanol extract of *M. triphylla* and a 50 mg/kg dose of diclofenac sodium on acetic acid-induced paw edema (Table 3).

#### 3.4.2. Effect of Duration of Treatment on Acetic Acid-Induced Paw Edema in *Wistar* Rats

Table 4 is a summary of the effect of duration of treatment on acetic acid-induced paw edema in rats.

**Figure 2:**
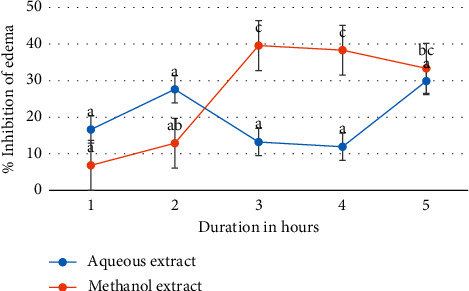
Effects of the duration of treatment on acetic acid-induced paw edema in *Wistar* rats. Values are presented as mean ± SEM. Means with different superscript letters along the same line are significantly different (two-way ANOVA followed by Tukey's test; *p* < 0.05).

Figure 2 further illustrates the onset and duration of action of *M. triphylla* root extracts in inhibiting acetic acid-induced paw edema in *Wistar* rats.

The duration of treatment did not significantly affect the efficacy of the aqueous extract of *M. triphylla* to inhibit acetic acid-induced paw edema in rats (*p* < 0.5; Table 4 and Figure 2). Conversely, the inhibition of acetic acid-induced paw edema in rats treated with the methanol extract was significantly higher (*p* < 0.05) after 3, 4, and 5 hours relative to after 1 hour (Table 4 and Figure 2). Moreover, there was no significant difference (*p* > 0.05) in the inhibition of acetic acid-induced paw edema in rats treated with the methanol extract after 2 hours relative to after 1 hour (Table 4 and Figure 2).

#### 3.4.3. Effect of Treatment and Duration on the Acetic Acid-Induced Paw Edema in *Wistar* Rats

There was no significant difference (*p* > 0.05) between the effect of low, intermediate, and high doses of the aqueous root extract of *M. triphylla* and the effect of diclofenac sodium on acetic acid-induced paw edema in rats after 1, 2, 3, 4, and 5 hours. However, the percentage inhibition of acetic acid-induced paw edema in rats treated with a low dose of the methanol root extract of *M. triphylla* after 1 hour was significantly lower (*p* < 0.05) than that in rats treated with a high dose after 3, 4, and 5 hours.

## 4. Discussion

The present study evaluated acute oral toxicity, phytochemical content, and the analgesic and anti-inflammatory properties of the aqueous and methanol root extracts of *M. triphylla*. The widespread use of *M. triphylla* as a source of traditional medicine throughout its distributional range suggests that the species is not taken at toxic dosages. However, the FDA and WHO emphasize the validation of efficacious and safe use of herbal therapies through the conduction of scientific-based studies [18, 19]. Therefore, rigorous toxicological and clinical studies of the leaves, fruits, bark, roots, and compounds isolated from the species are necessary [8] to determine their safer dose range [20].

In this study, the acute oral toxicity effects of both the aqueous and methanol root extracts of *M. triphylla* were determined using the Up-and-Down procedure described by Organization for Economic Cooperation and Development [11] (Document No. 425). This procedure has also been used in previous studies [21, 22]. There were no observable signs of toxicity in the experimental rats at the cut-off dose of 2000 mg/kg bw, suggesting that the extracts are nontoxic at therapeutic doses. This study disagrees with previous findings of Hamilton and Hamilton [23] and Dharani [24] that found out that the roots of *M. triphylla* have to be boiled for a long time to render them nontoxic. The toxicity here however could be due to repeated intake of the *M. triphylla* root porridge, hence requiring subacute or chronic toxicity assay.

According to Husna et al. [25], the absence of observable signs of toxicity and mortality in animals treated with a particular test dose implies that the LD_50_ is greater than the test dose. No morbidity or mortality was observed in any of the animals during the entire period of the study, suggesting that the LD_50_ of the aqueous and methanol root extracts of *M. triphylla* was above 2000 mg/kg bw. Previous studies have shown that the presence of toxic secondary metabolites like alkaloids is responsible for the adverse side effects reported when some plants or their products have been consumed [26]. These results, therefore, suggest that the studied plant extracts either lack the toxic metabolites or are present in too low concentrations to cause any observable signs of toxicity.

The phytochemical screening method of Harborne [13] and Kokate et al. [14] used in this study to qualitatively screen for phytoactive compounds in the plant extracts revealed the presence of cardiac glycosides, flavonoids, alkaloids, and phenols in both extracts, which is in agreement with previous studies [27]. Both extracts tested negative for terpenoids, steroids, and tannins. No saponins were present in the aqueous extract as opposed to the methanol extract that tested positive for saponins. This is in agreement with previous studies where the investigators established that not all phytochemicals may be found in plant parts [28].

Bioactive compounds such as flavonoids, alkaloids, saponins, phenols, and cardiac glycosides found in the extracts have been found to possess analgesic and anti-inflammatory properties in previous studies. Numerous studies have shown that alkaloids possess analgesic and anti-inflammatory properties [29, 30]. Flavonoids also show analgesic and anti-inflammatory activity by inhibiting prostaglandin synthetase, which in turn reduces prostaglandin synthesis and release [31–33]. Cardiac glycosides suppress hypersecretion of IL-8, a protein implicated in lung inflammation, thus inhibiting the activation of the NF-*β* signaling pathway. Phenols lower the expression and inhibit the function of iNOS. They also reduce the level of inflammatory mediators like TNF-*α* and prostaglandins [34].

The evaluation of the analgesic properties of the extracts of *M. triphylla* was determined using the acetic acid-induced writhing technique on *Swiss albino* mice of either sex. This technique is normally selected as a standard procedure to evaluate the peripheral analgesic efficacy of natural products and drugs and act by stimulating chemically induced stimulus [35]. The intraperitoneal injection of acetic acid induces the release of endogenous mediators such as prostaglandin, especially prostaglandin 2, histamine, bradykinin, and serotonin in peritoneal fluids. This produces peritoneal inflammation, which is associated with pain [36, 37]. This pain is characterized by abdominal muscle contractions, body elongation, and extension of forelimbs characterized as writhing whose frequency can be quantified [21]. Agents that inhibit or reduce the acetic acid-induced writhing frequency are considered as having an analgesic effect.

In this study, both the aqueous and methanol root extracts of *M. triphylla* demonstrated significant inhibition of the acetic acid-induced writhing in mice. Pain sensation in the acetic acid-induced writhing method is brought about by triggering localized inflammatory response resulting from the release of free arachidonic acid from tissue phospholipid [38] via cyclooxygenase and prostaglandin biosynthesis in peritoneal fluids [39]. Furthermore, other pain mediators like bradykinins and histamine are released from cells lining the peritoneal cavity and further help stimulate nociceptors. The increase in prostaglandin levels within the peritoneal cavity then enhances inflammatory pain by increasing capillary permeability [40]. These results suggest that the extracts were able to inhibit the prostaglandin synthesis, which is an inflammatory pain mediator. It can therefore be suggested that the flavonoids and alkaloids contained in the aqueous and methanolic root extracts of *M. triphylla* could be responsible for the analgesic effect of the extracts. This is similar to other results obtained in previous studies that have examined the analgesic activities of other medicinal plants [41].

The aqueous extract was more potent than the methanol extract throughout the dose levels. This suggests that the aqueous extract contains more active compounds responsible for the analgesic effect compared to the methanol extract at similar dose levels. The three dose levels of both the aqueous and methanol root extracts of *M. triphylla* produced a dose-dependent response to the acetic-induced pain. This kind of response was also observed by [21]. Peak analgesic effect was observed at a dose of 500 mg/kg in both extracts with the analgesic effect of both extracts being greater than that of the reference drug acetylsalicylic acid (75 mg/kg bw) at all dose levels except for methanol extract at a dose of 20 mg/kg bw. This could be explained by the fast metabolism and clearance of the active compounds that were in an insufficient concentration in the lower dose level of the methanol extract [1].

Inflammation is a normal, protective response to tissue injury caused by physical trauma, noxious chemicals, or microbiologic agents, which is a part of the host defense [2]. Some of the symptoms that characterize inflammation include the release of inflammatory mediators, vasodilation, increased blood flow, necrosis, tissue degeneration, and formation of exudates. Inflammation usually subsides on completion of the healing process, but sometimes it turns to severe, which may be fatal, leading to varying degrees of tissue injuries, diseases such as arthritis, and even death [3].

In this experiment, the anti-inflammatory properties of aqueous and methanol root extracts of *M. triphylla* in *Wistar* rats of either sex were investigated using acetic acid-induced paw edema. The subplantar injection of acetic acid induces the release of inflammatory mediators such as serotonin and histamine [37]. Therefore, there is increased vasculature permeability with greater vasodilation resulting in edema at the paw [37]. An increase in paw size is used to quantify the inflammatory response seen after acetic acid injection.

The results obtained in this study suggest a remarkable ability of the aqueous and methanol root extracts of *M. triphylla* in inhibiting acetic acid-induced paw edema in rats. NSAIDs alleviate inflammation by inhibiting the activity of phospholipase A_2_ and COX-2 [42]. Flavonoids, cardiac glycosides, phenols, and saponins have been reported in other studies to also have potent anti-inflammatory properties [33, 34]. Therefore, the flavonoids, cardiac glycosides, and phenols identified in both extracts could be responsible for the anti-inflammatory activity of the plant extracts by inhibiting prostaglandin synthesis in a similar manner to NSAIDs. The methanol extract had better percentage inhibition of inflammation than the aqueous extract of the same plant at the intermediate and high dose levels. A similar kind of response was also observed by [43, 44]. Apart from flavonoids, cardiac glycosides, and phenols, saponins were identified in the methanol extract but were absent in the aqueous extract. In previous studies, 3,4-seco-dammarane triterpenoid saponins isolated from *Cyclocarya paliurus* leaves have exhibited anti-inflammatory activity. This suggests that the saponins in the methanol root extract of *M. triphylla* could be responsible for the better potency of the methanol extract in inhibiting inflammation in comparison to the aqueous extract [45].

Peak anti-inflammatory effect was observed at a dose of 500 mg/kg in both extracts, with both extracts exhibiting a dose-dependent relationship. Similar results have also been observed by [21]. The anti-inflammatory effect of the aqueous extracts was not significantly different from the reference drug diclofenac sodium at all doses. This could imply that the concentration of the phytochemicals responsible for the anti-inflammatory activity of the aqueous extract was similar at all the studied doses. In the methanol extract, however, only the low methanol extract dose had a lower anti-inflammatory activity than the standard drug. This could be explained by the fast metabolism and clearance of the active compounds that were in an insufficient concentration in these lower dose levels of the extract [1].

The three doses of the aqueous extract and methanol extract together with their respective standard drugs (diclofenac sodium) achieved maximum anti-inflammatory activity in the second and third hours, respectively. This indicates a gradual but steady passive diffusion of the bioactive phytochemicals across the cell membrane into the site of inflammation [46]. This would suggest that the aqueous extract constituents diffused faster than the methanol extract constituents, hence achieving the maximal anti-inflammatory effect at the second hour rather than the third hour in the methanol extract. From the results, the aqueous extract at the three dose levels had low percentage inhibition of the acetic acid-induced paw edema in rats during the first hour. The methanol extract at the three dose levels had low percentage inhibition of the acetic acid-induced paw edema in rats during the first and second hours. This can be explained by the absence of prostaglandins in this early phase of inflammation since the treatments were working similarly to NSAIDs by inhibiting the biosynthesis of prostaglandins [42].

Previous studies have identified betaines and quaternary ammonium compounds such as 3-hydroxyproline betaine, glycine betaine, proline betaine, and 3-hydroxy-1,1-dimethyl pyrrolidinium from dried aerial parts and branches of *M. triphylla* [47]. Proline betaine has been shown to significantly suppress IL-1*β*-induced inflammation with decreased levels of inflammatory mediators and cytokines, including NO, PGE2, iNOS, COX-2, TNF-*α*, and IL-6 [48]. This compound has also been shown to dock at the COX-2 receptor using GOLD docking fitness and therefore inhibiting the activity of the COX-2 enzyme [49]. This suggests that proline betaine could be responsible for the analgesic and anti-inflammatory properties of the *M. triphylla* root extracts.

## 5. Conclusion

Based on the results obtained in this study, it was concluded that the aqueous and methanolic root extracts of *M. triphylla* have significant analgesic and anti-inflammatory effects, which may be due to the presence of phytochemicals such as flavonoids. In addition, both the aqueous and methanol root extracts of *M. triphylla* are nontoxic at therapeutic levels. Therefore, the aqueous and methanol *M. triphylla* root extracts may be used in the mitigation of pain and inflammation, as claimed in ethnomedicine.

However, isolation, characterization, and quantification of the specific phytochemical constituents in the *M. triphylla* root extracts with analgesic and anti-inflammatory activity is required. Moreover, future studies should focus on determining the specific analgesic and anti-inflammatory mechanism(s) of action of the *M. triphylla* root extracts at cellular and molecular levels. Additionally, apart from acute toxicity, evaluation of the subacute and chronic effects of the *M. triphylla* root extracts in experimental animal models should be done.

## Figures and Tables

**Table 1:** Phytochemical composition of *M. triphylla* root aqueous and methanol extracts.

Phytochemical	Aqueous extract	Methanol extract
Saponins	−	+
Alkaloids	+	+
Terpenoids	−	−
Flavonoids	+	+
Cardiac glycosides	+	+
Steroids	−	−
Phenols	+	+
Tannins	−	−

+: present; −: absent.

**Table 2:** Effect of *M. triphylla* root aqueous and methanol extract on acetic acid-induced writhing in Swiss albino mice.

Dose (mg/kg bw)	Aqueous root extract		Methanol root extract	
	Mean number of writhes	% writhing inhibition	Mean number of writhes	% writhing inhibition
20	13.00	85.31 ± 1.13^a^	15.80	81.84 ± 1.51^a^
100	5.80	93.43 ± 1.26^b^	10.00	88.43 ± 1.25^bc^
500	3.60	95.97 ± 1.16^b^	6.20	92.55 ± 1.20^c^
ASA (75 mg/kg bw)	16.60	81.08 ± 1.16^a^	15.60	82.17 ± 2.11^ab^
Physiological saline	88.20	0	88.00	0

Values are presented as mean ± SEM. Means with different superscript letters along the same column are significantly different (one-way ANOVA and Tukey's test; *p* < 0.05). ASA: acetylsalicylic acid.

**Table 3:** Summary of the effect of the aqueous and methanol extracts of *M. triphylla* on acetic acid-induced paw edema in *Wistar* rats.

Treatment (mg/kg bw)	% inhibition of edema	
	Aqueous extract	Methanol extract
Diclofenac sodium (50)	24.23 ± 7.78^a^	29.81 ± 5.76^bc^
20	9.11 ± 5.79^a^	7.57 ± 5.01^a^
100	17.48 ± 5.20^a^	19.77 ± 6.46^ab^
500	28.63 ± 6.21^a^	47.69 ± 6.57^c^

Values are presented as mean ± SEM. Means with different superscript letters along the same column are significantly different from each other (two-way ANOVA and Tukey's test; *p* < 0.05).

**Table 4:** Effects of the duration of treatment on acetic acid-induced paw edema in *Wistar* rats.

Duration	% inhibition of edema	
	Aqueous extract	Methanol extract
1 hour	16.63 ± 7.70^a^	6.83 ± 10.32^a^
2 hours	27.65 ± 3.12^a^	12.89 ± 4.86^ab^
3 hours	13.22 ± 6.52^a^	39.61 ± 3.87^c^
4 hours	11.92 ± 8.17^a^	38.34 ± 6.27^c^
5 hours	29.90 ± 8.57^a^	33.37 ± 7.05^bc^

Values are presented as mean ± SEM. Means with different superscript letters along the same column are significantly different (two-way ANOVA and Tukey's test *p* < 0.05).

## Data Availability

All data are available within the manuscript, and additional data are available from the corresponding authors upon request.
